# A phase 1 proof-of-concept study evaluating safety, tolerability, and biological marker responses with combination therapy of CTLA4-Ig and interleukin-2 in amyotrophic lateral sclerosis

**DOI:** 10.3389/fneur.2024.1415106

**Published:** 2024-06-10

**Authors:** Jason R. Thonhoff, David R. Beers, Weihua Zhao, Alireza Faridar, Aaron Thome, Shixiang Wen, Aijun Zhang, Jinghong Wang, Stanley H. Appel

**Affiliations:** Department of Neurology, Houston Methodist Neurological Institute, Houston Methodist Research Institute, Houston Methodist Hospital, Houston, TX, United States

**Keywords:** amyotrophic lateral scelerosis, neuroinflammation, interleukin-2 (IL-2), CTLA4-Ig, phase 1, clinical trial, oxidative stress, lipid perodixation

## Abstract

**Objective:**

To determine whether a combination therapy with abatacept (CTLA4-Ig) and interleukin-2 (IL-2) is safe and suppresses markers of oxidative stress, inflammation, and degeneration in ALS.

**Methods:**

In this open-label study, four participants with ALS received subcutaneous injections of low dose IL-2 (1 × 10^6^ IU/injection/day) for 5 consecutive days every 2 weeks and one subcutaneous injection of CTLA4-Ig (125 mg/mL/injection) every 2 weeks coinciding with the first IL-2 injection of each treatment cycle. Participants received a total of 24 treatment cycles during the first 48 weeks in this 56-week study. They were closely monitored for treatment-emergent adverse events (TEAEs) and disease progression with the ALSFRS-R. Phenotypic changes within T cell populations and serum biological markers of oxidative stress [4-hydroxynonenal (4-HNE) and oxidized-LDL (ox-LDL)], inflammation (IL-18), and structural neuronal degeneration [neurofilament light chain (Nf-L)] were assessed longitudinally.

**Results:**

CTLA4-Ig/IL-2 therapy was safe and well-tolerated in all four participants over the 56-week study. During the first 24 weeks, the average rate of change in the ALSFRS-R was +0.04 points/month. Over the 48-week treatment period, the average rate of change was −0.13 points/month with one participant improving by 0.9 points/month while the other three participants experienced an average decrease of −0.47 points/month, which is slower than the average − 1.1 points/month prior to initiation of therapy. Treg suppressive function and numbers increased during treatment. Responses in the biological markers during the first 16 weeks coincided with minimal clinical progression. Mean levels of 4-HNE decreased by 30%, ox-LDL decreased by 19%, IL-18 decreased by 23%, and Nf-L remained the same, on average, in all four participants. Oxidized-LDL levels decreased in all four participants, 4-HNE and IL-18 levels decreased in three out of four participants, and Nf-L decreased in two out of four participants.

**Conclusion:**

The combination therapy of CTLA4-Ig and IL-2 in ALS is safe and well-tolerated with promising results of clinical efficacy and suppression of biomarkers of oxidative stress, neuroinflammation and neuronal degeneration. In this open-label study, the efficacy as measured by the ALSFRS-R and corresponding biomarkers suggests the therapeutic potential of this treatment and warrants further study in a phase 2 double-blind, placebo-controlled trial.

**Clinical trial registration:**

ClinicalTrials.gov, NCT06307301.

## Introduction

Dysregulation of the immune system promotes disease progression in amyotrophic lateral sclerosis (ALS) (1, 2). Regulatory T-lymphocyte (Treg) numbers and suppressive functions are reduced in patients with ALS, and decreased Treg suppressive function is associated with greater burden of disease, more rapid progression, and shortened survival (3). To offset the negative impact of dysfunctional neuroprotective Tregs in ALS patients, two clinical trials with autologous infusions of expanded Tregs combined with subcutaneous low-dose IL-2 were carried out (4, 5). Removing Tregs from the milieu of markedly proinflammatory macrophages and expanding them *ex vivo* restored the ability to suppress responder T cell proliferation and cytokine release from activated macrophages (3, 6). Autologous infusions of these expanded highly suppressive Tregs were safe and well tolerated in phase 1 and phase 2a clinical trials in ALS (4, 5). Treg numbers and suppressive function were enhanced in both trials. However, the COVID-19 pandemic limited the number of participants in the planned first 6-month placebo-controlled portion of the phase 2a trial. In the second 6-month open-label portion of the trial, slowing of clinical progression to an average of −0.45 points/month per the ALSFRS-R was noted in six out of the eight participants. Two of the eight participants were unresponsive to the therapy and exhibited marked elevation of serum markers of oxidative stress (oxidized-LDL and the lipid peroxide, 4-HNE) and inflammation (IL-17). The six participants who responded to the therapy had levels of these markers within the normal range (5).

These pilot trials suggest that the infused expanded Tregs did not retain their neuroprotective functions in the presence of dramatically increased levels of oxidative stress. Expanded Tregs are not end-stage differentiated and can express Th17 in the presence of IL-6 (7, 8). IL-6 in turn is well-recognized to be released from activated proinflammatory macrophages, which can also promote the production of 4-HNE (9–11). We hypothesize that the activation of proinflammatory myeloid cells contributes to the loss of Treg numbers and function in ALS and could limit the efficacy of therapies aimed to enhance the neuroprotective functions of Tregs. Our preliminary *in vitro* data suggest that activated proinflammatory myeloid cells enhance the loss of Treg numbers and function. Suppressing activated myeloid cells with CTLA4-Ig (Abatacept/Orencia ®), which binds to CD80 and CD86 and prevents interaction with CD28, in combination with subcutaneous low dose IL-2 could improve Treg survival and function in ALS.

Subcutaneous administration of low-dose IL-2 has been safely administered in many human trials with minimal side effects (12, 13). Abatacept is an FDA-approved medication that has been used as a monotherapy or concomitantly with other anti-inflammatory drugs to modulate inflammation in autoimmune disorders. To our knowledge, CTLA4-Ig has never been evaluated in ALS. In the present open-label study, we evaluate the safety and tolerability of CTLA4-Ig together with subcutaneous low-dose IL-2 administration in four participants with ALS and monitor biological markers of oxidative stress (oxidized-LDL and 4-HNE), proinflammatory cytokine release (IL-18), and the cytoskeletal component (Nf-L).

## Methods

### Primary research question

Is the combination therapy of subcutaneous CTLA4-Ig and subcutaneous low-dose IL-2 administration safe and tolerable in participants with ALS? This study is an open label phase 1 proof of concept trial, which provides class IV evidence that CTLA4-Ig/IL-2 combination therapy is safe and well-tolerated over 48 weeks of treatment.

### Standard protocol approvals, registrations, and patient consents

Approval from the Food and Drug Administration (FDA) was not required to conduct this study. Approval from the Institutional Review Board at Houston Methodist Hospital was obtained prior to study initiation. Written informed consent was obtained prior to enrollment. The study was registered on ClinicalTrials.gov (NCT06307301).

### Study design and participant selection criteria

This study was conducted at the Houston Methodist Neurological Institute. Participants with and without a family history of ALS, and with differing sites of symptom onset and rates of disease progression, were recruited from Houston Methodist Hospital’s MDA/ALSA ALS clinic. Four participants were enrolled in the trial (Table 1), treated and followed between December 2021 and July 2023. Healthy controls were recruited under an IRB-approved protocol through Houston Methodist Hospital for biomarker sample collection. Samples from 23 healthy controls were utilized in this study. Of the 23 controls, nine were men (39%) and 14 were women (61%), and the average age was 60.7 ± 7.3 years (mean ± standard deviation).

**Table 1:** Participant baseline characteristics.

Participant no.	1	2	3	4
Age (years)	47	54	57	84
Sex	Female	Male	Female	Female
Race	Hispanic	White	White	Hispanic
Initial weight (kg)	121.1	110.7	50.0	50.2
Associated genetic mutation	C9ORF72	No	No	No
Site of symptom onset	Limb	Limb	Bulbar	Bulbar
Time from symptom onset to diagnosis (months)	4	4	5	18
Time from symptom onset to first treatment (months)	31	13	10	27
Riluzole use at study entry	No	Yes	Yes	Yes
Edaravone use at study entry	No	No	No	No
Non-invasive ventilation use at study entry	No	Yes	Yes	No
Vital capacity (L) just prior to first treatment	3.74	1.94	2.96	2.24
Vital capacity (% predicted) just prior to 1st treatment	102.7	41	94.6	110
MIP (cm H_2_O) just prior to first treatment	127	28	54	27
ALSFRS-R just prior to first treatment	30	27	38	39
AALS score just prior to first treatment	51	72	49	62
Disease progression rate at first treatment (ALSFRS-R points/month) calculated by the change in ALSFRS-R from the last ALS clinic visit	−1.6	−1.0	−1.0	−0.7

### Administration of subcutaneous CTLA4-Ig and IL-2

During this 56-week study, participants received a total of 24 subcutaneous injections of CTLA4-Ig (Abatacept, Bristol Myers Squibb; 125 mg/mL) and 23 5-day courses of daily subcutaneous injections of human recombinant IL-2 (Clinigen; 1 × 10^6^ units/day). On week 0 day 1, participants received CTLA4-Ig only. On week 2 day 1, participants received CTLA4-Ig followed by IL-2 and then four more consecutive days of IL-2, and this regimen was continued every 2 weeks throughout the 48-week treatment period. Participants then underwent an 8-week follow up period with no CTLA4-Ig/IL-2 treatment.

### Assessing Treg numbers and suppressive functions

Peripheral blood for assessing Treg numbers and suppressive function was drawn at baseline and then every 4 weeks throughout the first 24-week of the study immediately prior to initiating each treatment cycle. During the second 24-week portion of the study, peripheral blood for these assessments was drawn every 12 weeks and finally at week 52. The percentage of CD4^+^CD25^+^FOXP3^+^ Tregs within the total CD4^+^ population and all other T cell markers were assessed by flow cytometry (3). Treg suppressive function on the proliferation of autologous responder T lymphocytes was assessed by [^3^H]-thymidine incorporation (3).

### Clinical evaluations

Revised ALS Functional Rating Scale (ALSFRS-R) and Appel ALS rating scale (AALS) (14) measurements were performed at baseline (week 0 day 1) and then every 4 weeks immediately prior to treatment throughout 24 and 48 weeks of treatment and 8-week of follow up with the final assessment occurring on week 56. Participants also returned to clinic every 2 weeks to receive injections of CTLA4-Ig and IL-2 under observation during the 48-week treatment period, and to pick up the four remaining IL-2 syringes to complete each treatment cycle, which they kept refrigerated at home. Participants were asked about adverse events at each encounter.

### Assessing biological markers of oxidative stress, inflammation, and structural degeneration

Peripheral blood for assessing biological markers was collected at baseline and then every 2 weeks throughout the study immediately prior to initiating each treatment cycle. Markers of oxidative stress (oxidized-LDL/ox-LDL and 4-hydroxynonenal/4-HNE), inflammation (interleukin-18/IL-18), and structural degeneration (neurofilament light chain/Nf-L) were assayed by ELISA (4-HNE, Cell Biolabs, Inc., Cat# STA-838; ox-LDL, Mercodia, Cat# 10-1143-01; IL-18, Fisher, Cat# KHC0181; Nf-L, MyBioSource, Cat# MBS9399603) to determine their concentrations in sera of participants (*n* = 4) and healthy controls (*n* = 23) according to manufacturer’s instructions. Levels of soluble CD25 (sCD25) were also assayed by ELISA (R&D Systems, Cat# DR2A00) using sera from participants (*n* = 4) and healthy controls (*n* = 23) according to the manufacturer’s instructions.

### Statistical analysis

Changes in Treg suppressive function and flow parameters at baseline, week 16, week 24, week 48, and week 52 (6-week post-treatment period) were determined by a one-way ANOVA with *post-hoc* Tukey test using GraphPad Prism 7 software. Correlation between changes in the ALSFRS-R and AALS was determined by Spearman’s correlation using GraphPad Prism 7 software and depicted by Spearman’s rho (ρ). Two-tailed *p* values less than 0.05 were considered significant. Due to the small numbers and high variability among participants, descriptive statistics were used to observe changes in the ALSFRS-R and AALS as well as changes in biological markers throughout the course of the study.

### Data availability

Individual de-identified participant data not published within the article including clinical assessments, biological marker results, and Treg percentage and suppressive function results will be shared by request from any qualified investigator.

## Results

### Baseline clinical characteristics

The demographics and clinical characteristics at baseline are shown for each participant in Table 1. Three participants were women, participant #1 had mutant C9ORF72-mediated ALS, and three participants had respiratory insufficiency according to a qualifying low maximal inspiratory pressure (MIP) value (≤ 60 cm H_2_O), two of which were already being treated with noninvasive ventilation. Disease progression rates calculated by serial ALSFRS-R assessments prior to baseline ranged from −0.7 to −1.6 points/month, averaging −1.1 points/month in all four participants.

### Safety and tolerability of CTLA4-Ig/IL-2 treatment

A summary of the treatment-emergent adverse events (TEAEs) in all participants is shown in Table 2. All TEAEs were considered mild and there were no serious adverse events. Two participants experienced falls commonly related to the progression of ALS. Two participants contracted COVID-19 infections that were self-limiting and required no treatment. COVID-19 infections were rampant during the time this study was conducted due to the COVID-19 pandemic. The two participants recovered well while on CTLA4-Ig/IL-2 treatment indicating no impairment of immune reactivity to their COVID-19 infections. One participant had persistent injection site reactions. Tolerability as defined by the percentage of participants who completed the study was 100%.

**Table 2:** Treatment-emergent adverse events (TEAEs) by system organ class.

System organ class/Preferred term	Abatacept/IL-2
	*N* = 4 (%)
Any TEAE	4 (100)
Gastrointestinal disorders	
Diarrhea	1 (25)
Nausea	1 (25)
General disorders and administration site conditions	
Injection site erythema	1 (25)
Infections	
Coronavirus infection	2 (50)
Injury complications	
Ankle swelling	2 (50)
Fall	2 (50)
Head contusion	2 (50)
Investigations	
Hypokalemia	1 (25)
Musculoskeletal disorders	
Muscle spasms	1 (25)
Myalgia	1 (25)
Respiratory disorders	
Nasal congestion	1 (25)

### Treg number and suppressive function increase in response to CTLA4-Ig/IL-2 treatment

**Figure 1:**
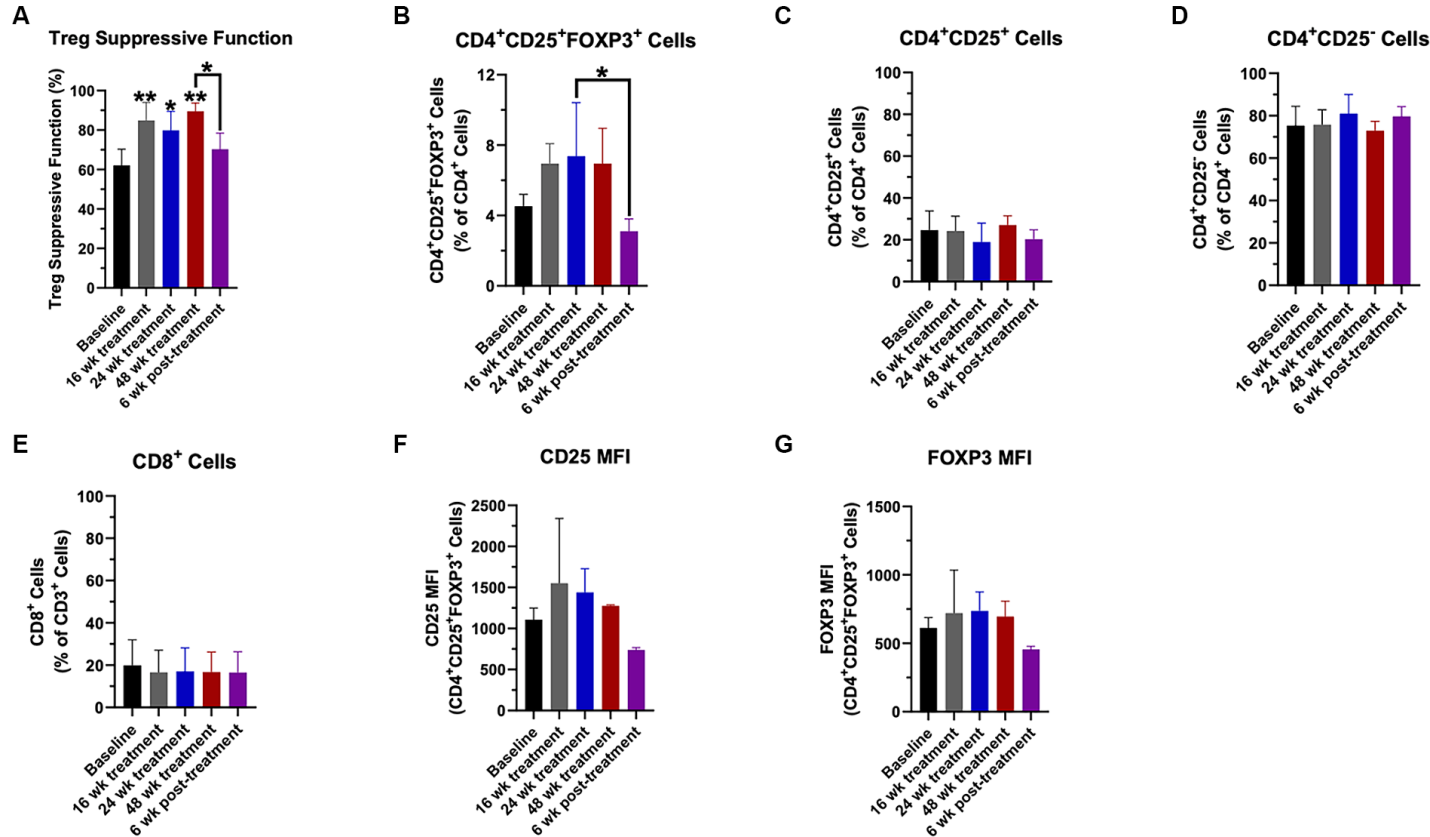
Treg numbers and suppressive function increased with CTLA4-Ig/IL-2 treatment throughout the 48-week treatment period. Changes in Treg suppressive function **(A)**, the percentage of CD4^+^CD25^+^FOXP3^+^ cells **(B)**, the percentage of CD4^+^CD25^+^ cells **(C)**, the percentage of CD4^+^CD25^−^ cells **(D)**, and the percentage of CD8^+^ cells **(E)** throughout the study are shown. Changes in CD25 protein expression **(F)** and FOXP3 protein expression **(G)** in the CD4^+^CD25^+^FOXP3^+^ population by mean fluorescent intensity (MFI) throughout the study are shown. Data were analyzed by a one-way ANOVA with *post-hoc* Tukey test and *p* values less than 0.05 were considered significant. Data are expressed as mean ± SD. ^*^p < 0.05, ^**^p < 0.01.

Regulatory T-lymphocyte suppressive function was increased compared to baseline at weeks 16, 24, and 48 (Figure 1A). Six weeks following the final course of CTLA4-Ig/IL-2 treatment on week 46, the Treg suppressive function returned to the baseline level (Figure 1A). The percentage of Tregs (CD4^+^CD25^+^FOXP3^+^ cells) within the CD4^+^ population trended up throughout the study (Figure 1B). Following the 6-week washout period, the Treg percentage decreased compared to its peak value at week 24 (Figure 1B). There were no differences in the other T cell flow parameters assessed throughout the study including the percentage of CD4^+^CD25^+^ cells (Figure 1C), CD4^+^CD25^−^ cells (Figure 1D), and CD8^+^ cells (Figure 1E). There were also no differences in the amount of CD25 and FOXP3 protein expression on Tregs, although there was an increasing trend (Figures 1F,G, respectively).

### Clinical progression stabilized during 24 and 48 weeks of CTLA4-Ig/IL-2 treatment

**Figure 2:**
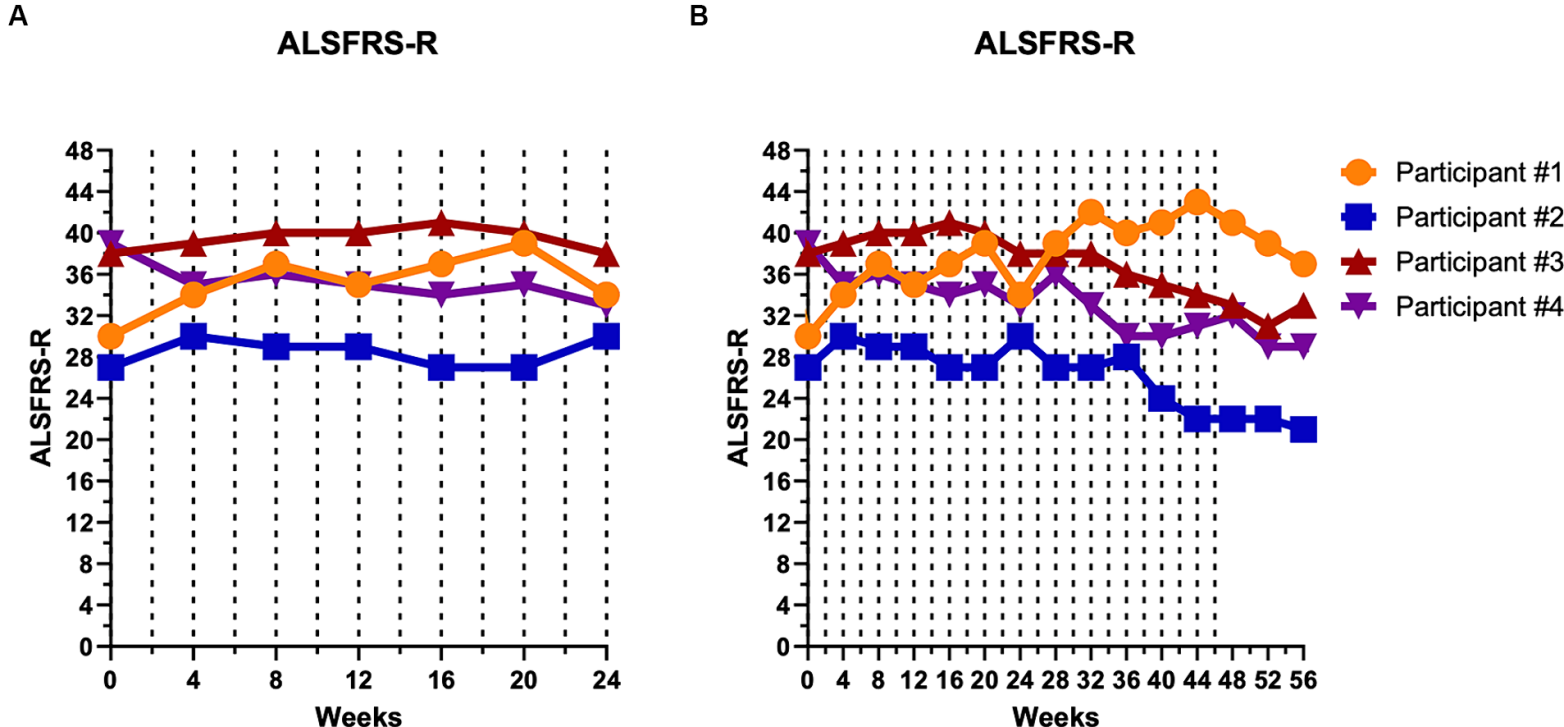
Disease progression remained relatively unchanged during the first 24 weeks of CTLA4-Ig/IL-2 treatment. Dashed vertical lines represent either a CTLA4-Ig injection alone (week 0) or CTLA4-Ig injection simultaneously with 5 consecutive days of IL-2 injections (week 2–48). Clinical progression is depicted by the ALSFRS-R over the first 24 weeks **(A)** and over the entire 56-week study **(B)**. At week 24, participant #1 improved by four points, participant #2 improved by three points, participant #3 was unchanged, and participant #4 decreased by six points. Throughout the entire 48-week treatment period, participant #1 improved by 11 points, participant #2 decreased by five points, participant #3 decreased by five points, and participant #4 decreased by seven points.

For all four participants, the mean rate of change in the ALSFRS-R over the first 24 weeks was stable (+0.04 points/month). During this time period, the ALSFRS-R improved by four points in participant #1, improved by three points in participant #2, was unchanged in participant #3, and decreased by six points in participant #4 (Figure 2A). Participant #4 experienced a four-point decrease at week 4, but then remained relatively stable from weeks 4 to 24.

Over the 48-week treatment period, the average rate of change in the ALSFRS-R was −0.13 points/month. Over the entire 48 weeks, participant #1 had an 11-point improvement while participant #2 decreased by five points, participant #3 decreased by five points, and participant #4 decreased by seven points (Figure 2B). Even when excluding participant #1, who had improved, participants #2, 3, and 4 decreased by −0.47 points/month, a rate well below the −1.1 points/month progression rate prior to initiating therapy. Disease progression per the AALS, an objective assessment, mirrored the ALSFRS-R with participants showing relative stability during the first 24 weeks of the study and then gradual deterioration in participants #2, 3, and 4 (data not shown). The ALSFRS-R and AALS showed strong correlation in all participants throughout the study (ρ = −0.754, *p* < 0.001).

### Biological markers are suppressed during the first 16-weeks of CTLA4-Ig/IL-2 treatment

**Figure 3:**
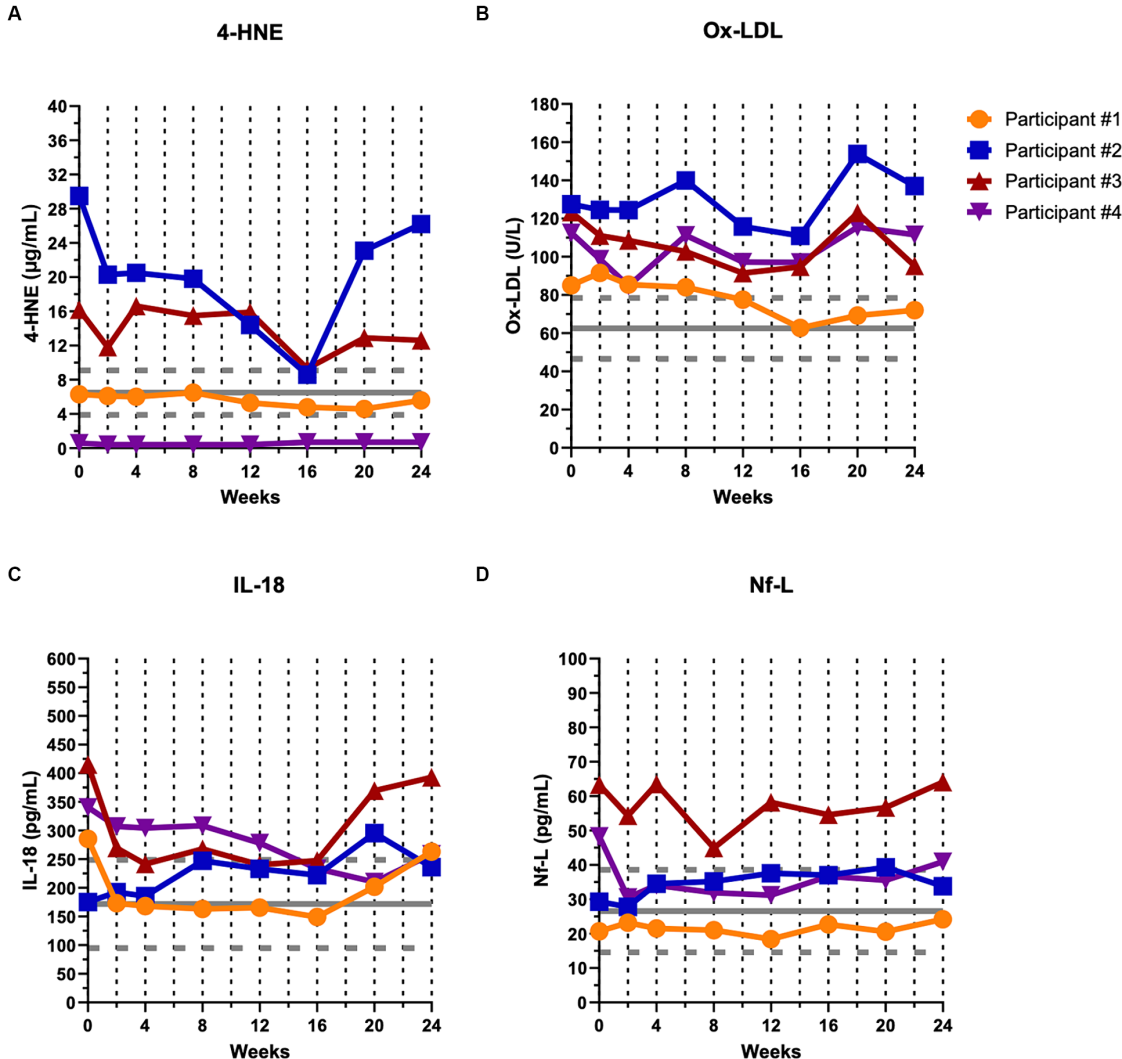
Biological markers of oxidative stress, inflammation, and structural degeneration showed a decreasing trend during the first 16 weeks of CTLA4-Ig/IL-2 treatment. Dashed vertical lines represent either a CTLA4-Ig injection alone (week 0) or CTLA4-Ig injection simultaneously with 5 consecutive days of IL-2 injections (week 2–24). The horizontal gray solid line and dashed lines indicate healthy control mean ± SD (*n* = 23) for each marker, respectively. Changes in the levels of two markers of oxidative stress, 4-HNE **(A)** and ox-LDL **(B)**; a marker of inflammation, IL-18 **(C)**; and a marker of neuronal structural degeneration, Nf-L **(D)** are shown over the first 24 weeks of the study in all four participants.

Biological markers of oxidative stress (Figure 3A, 4-HNE and Figure 3B, ox-LDL), inflammation (Figure 3C, IL-18), and structural degeneration (Figure 3D, Nf-L) are depicted at baseline and throughout the first 24-week of the study (Figure 3), and then throughout the remaining course of the study (Supplementary Figures S1A–D). The mean ± SD for healthy controls is shown in gray for each marker. Participants #2 and 3 had elevated levels of 4-HNE compared to controls whereas participant #1 and #4 showed levels within the control range (Figure 3A). Participants #1, 2, and 3 showed a decreasing trend of 4-HNE during the first 16-week of treatment before levels appeared to rise again (Figure 3A; Table 3). All four participants showed levels of oxidized-LDL above control levels (Figure 3B), and all four participants showed a decreasing trend during the first 16-week of treatment, especially participant #1, before levels began to rise again (Figure 3B; Table 3). Participants #1, 3, and 4 showed levels of IL-18 higher than controls at baseline whereas participant #2 had levels within the control range (Figure 3C). IL-18 levels decreased in participants #1, 3, and 4 during the first 16-week of treatment whereas participant #2 showed increasing levels during that early time period (Figure 3C; Table 3). Finally, participants #3 and 4 exhibited elevated levels of Nf-L at baseline whereas participants #1 and 2 were within control levels (Figure 3D). Participants #3 and 4 also showed decreasing trends in Nf-L for 16-weeks after initiating treatment while levels in participants #1 and 2 remained unchanged and increased, respectively (Figure 3D; Table 3).

**Table 3:** Changes in biological marker levels over the first 16 weeks of CTLA4-Ig/IL-2 treatment.

Marker	Participant	Week 0	Week 4	Week 8	Week 12	Week 16	Trend (%)
4-HNE	#1	6.3	6.0	6.5	5.3	4.8	−23.8
(μg/mL)	#2	29.5	20.5	19.8	14.4	8.6	−70.8
	#3	16.2	16.6	15.5	15.9	9.3	−42.6
	#4	0.6	0.4	0.4	0.4	0.7	16.7
Average							−30.1
Ox-LDL	#1	85.0	85.4	84.0	77.5	62.7	−26.2
(U/L)	#2	127.5	124.4	139.9	115.8	110.9	−13.0
	#3	123.3	108.6	102.7	91.4	94.8	−23.1
	#4	112.5	84.7	111.2	97.2	97.0	−13.8
Average							−19.0
IL-18	#1	285.5	168.0	163.0	165.5	149.3	−47.7
(pg/mL)	#2	175.3	185.5	247.2	233.1	222.2	26.8
	#3	415.0	241.5	267.9	240.0	247.4	−40.4
	#4	340.6	304.4	308.6	278.1	233.6	−31.4
Average							−23.2
Nf-L	#1	20.7	21.5	21.0	18.4	22.7	9.7
(pg/mL)	#2	29.3	34.5	35.2	37.6	37.0	26.3
	#3	63.3	63.5	44.9	58.2	54.6	−13.7
	#4	48.3	34.1	31.9	31.2	36.7	−24.0
Average							−0.5

## Discussion

Amyotrophic lateral sclerosis is a devastating condition with only modest disease-modifying therapies available. Additional therapies are clearly required to address the tremendous unmet need. The present trial used a combination immunomodulatory therapy to suppress neuroinflammation and attempt to slow disease progression. Four study participants with ALS received a 5-day treatment course with subcutaneous injections of CTLA4-Ig (day 1) simultaneously with low dose IL-2 (days 1–5) every 2 weeks over a 48-week period. The combination therapy was safe and well-tolerated in all participants. Disease progression appeared to stabilize in all participants during the first 24-weeks of the study, changing overall by +0.04 points/month per the ALSFRS-R. Over 48 weeks, the overall change in all participants was −0.13 points/month. Even excluding participant #1 with the hexanucleotide repeat expansion in *C9ORF72* who improved over the 48 weeks, participants #2, 3, and 4 decreased at an average rate of −0.47 points/month by the ALSFRS-R. These results are extremely promising as an average change of ALSFRS-R by 1 point/month would have been expected. In the two most recent FDA approvals for ALS, the estimated changes over 24-weeks were − 1.24 points/month for sodium phenylbutyrate-taurursodiol and − 0.84 points/month (−5.01 points over 6 months) for edaravone per the ALSFRS-R (15, 16). In a follow up phase 3 PHOENIX trial (ClinicalTrials.gov, NCT05021536) evaluating sodium phenylbutyrate-taurursodiol in ALS, the manufacturer reported no difference between treatment and placebo over a 24-week period, however.

Regulatory T-lymphocyte function also increased in all participants and remained elevated until the treatment effects had washed out by 6 weeks following the final treatment course. The percentage of Tregs also trended up during the treatment period before returning to baseline levels after the 6-week post-treatment period. Biological markers of oxidative stress, inflammation, and structural degeneration were also suppressed by the CTLA4-Ig/IL-2 treatment during the first 16 weeks. Elevated levels in these markers have been associated with more rapid disease progression, and decreased levels associated with slower disease progression. The decrease in the biological markers during the first 16 weeks is in accord with the observed minimal to no disease progression.

In ALS, the neuroprotective functions of Tregs become impaired as acceleration of disease progression ensues (3, 17). Macrophages/microglia adopt a proinflammatory phenotype that promotes Treg dysfunction (18). Myeloid-mediated inflammatory cytokines, oxidative stress and lipid peroxides are increased in ALS and may promote neuronal injury and cell death (1, 10). Further, protein adducts between superoxide dismutase 1 (SOD1) and 4-HNE as well as TDP-43 and 4-HNE, the latter resulting in aberrant cytosolic localization of TDP-43, have been identified in ALS (19, 20). These adducts propagate protein aggregation, which is a key component of the pathogenesis. Thus, therapies that ameliorate lipid peroxidation and the formation of 4-HNE could potentially slow disease progression, making 4-HNE a promising biological marker and surrogate marker of efficacy for immunotherapies. Treatment with low-dose IL-2 alone expands endogenous Tregs and suppresses the proliferation of responder T cells (12, 13). Expanded endogenous Tregs also suppress proinflammatory myeloid cells (21). However, Tregs become dysfunctional in the presence of myeloid cells with a markedly increased proinflammatory phenotype, even converting into a neurotoxic Th17 phenotype.

In a prior phase 1 study assessing treatment with autologous expanded Treg infusions in combination with low-dose subcutaneous IL-2, the participants experienced stabilization of disease and suppression of several markers of oxidative stress and acute phase proteins during periods of Treg infusions but showed a rebound elevation in these markers associated with clinical deterioration during periods of low-dose IL-2 treatment alone (4, 22). In addition, assessment of low-dose IL-2 therapy in people with ALS in Europe improved Treg numbers but had a minimal effect on disease progression (13). The MIROCALS phase 2b study (clinicaltrials.gov, NCT03039673) showed a modest decrease in the risk of death that was not statistically significant in a larger population of people with ALS treated with low-dose IL-2. Treatments that only enhance Tregs without targeting the proinflammatory myeloid cells that promote Treg dysfunction may not have durable effects on disease progression. In our Phase 2A study assessing the combination therapy of autologous expanded Tregs and low-dose IL-2, the two participants who appeared to have no response to the treatment exhibited very high levels of oxidative stress (5). However, in the present study, even though participant #2 also had very high levels of 4-HNE, he did respond to the combination of CTLA4-Ig with IL-2. The difference between the Phase 2A trial and the present trial was suppressing inflammatory myeloid cells with CTLA4-Ig in combination with IL-2.

In the present study, treatment with CTLA4-Ig/IL-2 suppressed myeloid-mediated oxidate stress (4-HNE and ox-LDL) and proinflammatory cytokines (IL-18) in addition to enhancing Treg suppressive function. The suppression of 4-HNE, ox-LDL and IL-18 occurred early in the treatment course, and Nf-L, a well-accepted marker of neuronal structural degeneration and disease progression in ALS, was also suppressed early. Suppression of these markers was associated with relatively stable disease for at least 24 weeks in all participants. This study also provides evidence that oxidative stress and inflammation may be differentially expressed in patients with ALS.

Although this study included a low number of participants and lacked blinding and placebo controls, slowing of disease progression was quite marked during the first 24-week of the study, and even over the 48-week compared to expected rates of progression as shown by the two separate scales monitoring disease progression, the ALSFRS-R and AALS, which correlated strongly with each other in all participants throughout the study. The underlying mechanism for increased clinical progression rates that were observed in three of four participants beginning from week 36 needs further exploration. Possible explanations include underdosing of the CTLA4-Ig and/or IL-2 resulting in progressive myeloid activation over time; the development of IL-2 anti-drug antibodies over time that subsequently reduced the activation of the CD25 receptor on Tregs; or increasing levels of soluble CD25 (sCD25) over time that decreased the bioavailable IL-2 concentration. Increasing levels of sCD25 in the sera over time was not observed in the four participants in this study (data not shown). The reason for participant #1’s clinical improvement throughout the 48-week is also unknown. Mutations in *C9ORF72* have been shown to increase microglia proinflammatory activity, which likely represents a major pathogenic mechanism in these particular patients (17), and may well be responsive to the combination therapy of CTLA4-Ig and IL-2. Whether patients with *C9ORF72*-ALS causative gene mutations may be more responsive to this treatment needs further investigation. Participant #1 also exhibited lower levels of all four markers that were maintained within the control ranges for most of the duration of the study. Whether patients with high levels of oxidative stress, inflammation and/or structural degeneration at baseline show a durable response with combination therapy of CTLA4-Ig/IL-2 also needs further investigation. Answers to these questions may help stratify patients in future clinical trials and develop personalized medicine strategies depending on the activated inflammatory pathways.

These data suggest that the combination of CTLA4-Ig and low dose IL-2 provides a promising approach to slowing progression in ALS; biomarkers of oxidative stress, proinflammatory cytokines, and cytoskeletal constituents may provide a guide to individual clinical outcomes. A phase 2, randomized, placebo-controlled trial over a prolonged period is needed to test the clinical safety, tolerability, and potential efficacy of different doses. The goal of future studies is to determine whether optimized doses of CTLA4-Ig/IL-2 slow disease progression, and whether there are populations of patients with specific patterns of biological markers that respond more favorably to immunomodulatory treatment.

## Data availability statement

The original contributions presented in the study are included in the article/Supplementary material; further inquiries can be directed to the corresponding author.

## Ethics statement

The studies involving humans were approved by Institutional Review Board at Houston Methodist Hospital. The studies were conducted in accordance with the local legislation and institutional requirements. The participants provided their written informed consent to participate in this study.

## Author contributions

JT: Conceptualization, Data curation, Formal analysis, Funding acquisition, Investigation, Methodology, Supervision, Writing – original draft. DB: Data curation, Formal analysis, Writing – review & editing. WZ: Data curation, Formal analysis, Writing – review & editing. AF: Conceptualization, Writing – review & editing. AT: Conceptualization, Writing – review & editing. SW: Data curation, Formal analysis, Writing – review & editing. AZ: Data curation, Formal analysis, Writing – review & editing. JW: Data curation, Formal analysis, Writing – review & editing. SA: Conceptualization, Formal analysis, Funding acquisition, Methodology, Supervision, Writing – review & editing.

## Supplementary material

SUPPLEMENTARY FIGURE S1 Biological markers of oxidative stress, inflammation and structural degeneration throughout 48 weeks of CTLA4-Ig/IL-2 treatment. Dashed vertical lines represent CTLA4-Ig injection simultaneously with 5 consecutive days of IL-2 injections (week 24–46). The horizontal gray solid line and dashed lines indicate healthy control mean ± SD (*n* = 23) for each marker, respectively. Changes in the levels of two markers of oxidative stress, 4-HNE **(A)** and ox-LDL **(B)**; a marker of inflammation, IL-18 **(C)**; and a marker of neuronal structural degeneration, Nf-L **(D)** are shown from week 24 to the end of the 56-week study in all four participants.
