# Supplementary figures and images for: A phase 1 proof-of-concept study evaluating safety, tolerability, and biological marker responses with combination therapy of CTLA4-Ig and interleukin-2 in amyotrophic lateral sclerosis
Source: Front Neurol. 2024 Jun 10;15:1415106. doi: 10.3389/fneur.2024.1415106 (PMC11195540; doi:10.3389/fneur.2024.1415106)

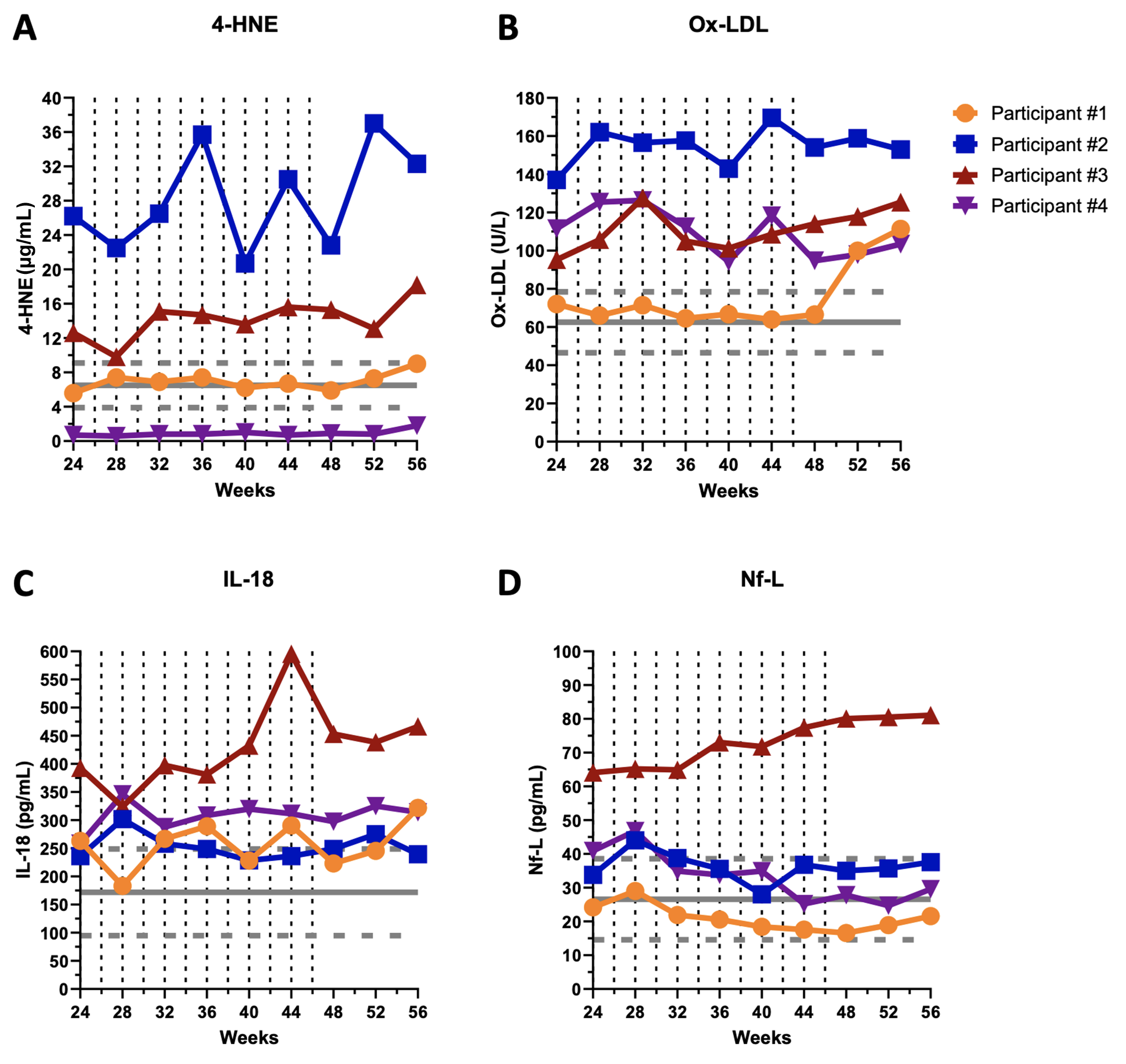

Supplement: SUPPLEMENTARY FIGURE S1 — Biological markers of oxidative stress, inflammation and structural degeneration throughout 48 weeks of CTLA4-Ig/IL-2 treatment. Dashed vertical lines represent CTLA4-Ig injection simultaneously with 5 consecutive days of IL-2 injections (week 24–46). The horizontal gray solid line and dashed lines indicate healthy control mean ± SD (n = 23) for each marker, respectively. Changes in the levels of two markers of oxidative stress, 4-HNE (A) and ox-LDL (B); a marker of inflammation, IL-18 (C); and a marker of neuronal structural degeneration, Nf-L (D) are shown from week 24 to the end of the 56-week study in all four participants. [file Image_1.TIF]
